# Bulk chemical composition contrast from attractive forces in AFM force spectroscopy

**DOI:** 10.3762/bjnano.12.5

**Published:** 2021-01-18

**Authors:** Dorothee Silbernagl, Media Ghasem Zadeh Khorasani, Natalia Cano Murillo, Anna Maria Elert, Heinz Sturm

**Affiliations:** 1 BAM Bundesanstalt für Materialforschung und -prüfung, Unter den Eichen 87, 12205 Berlin, Germany; 2 TU Berlin, IWF, Pascalstr. 8–9, 10587 Berlin, Germany

**Keywords:** AFM force spectroscopy, composites, principle component analysis, structure–property correlation, van der Waals forces

## Abstract

A key application of atomic force microscopy (AFM) is the measurement of physical properties at sub-micrometer resolution. Methods such as force–distance curves (FDCs) or dynamic variants (such as intermodulation AFM (ImAFM)) are able to measure mechanical properties (such as the local stiffness, *k*_r_) of nanoscopic heterogeneous materials. For a complete structure–property correlation, these mechanical measurements are considered to lack the ability to identify the chemical structure of the materials. In this study, the measured attractive force, *F*_attr_, acting between the AFM tip and the sample is shown to be an independent measurement for the local chemical composition and hence a complete structure–property correlation can be obtained. A proof of concept is provided by two model samples comprised of (1) epoxy/polycarbonate and (2) epoxy/boehmite. The preparation of the model samples allowed for the assignment of material phases based on AFM topography. Additional chemical characterization on the nanoscale is performed by an AFM/infrared-spectroscopy hybrid method. Mechanical properties (*k*_r_) and attractive forces (*F*_attr_) are calculated and a structure–property correlation is obtained by a manual principle component analysis (mPCA) from a *k*_r_/*F*_attr_ diagram. A third sample comprised of (3) epoxy/polycarbonate/boehmite is measured by ImAFM. The measurement of a 2 × 2 µm cross section yields 128 × 128 force curves which are successfully evaluated by a *k*_r_/*F*_attr_ diagram and the nanoscopic heterogeneity of the sample is determined.

## Introduction

The mechanical properties of small volumes of materials can be measured using various atomic force microscopy (AFM) methods. The well-established force–distance curve (FDC) method is the most fundamental force spectroscopy experimental setup which yields local mechanical properties with a lateral resolution between 500 nm and 1 µm [1–3]. Recent developments have aimed to increase the lateral resolution of force spectroscopy by implementing dynamic methods. This has resulted in methods such as force modulation [4], bimodal mode [5], pulsed-force mode [6] or peak force [7], and intermodulation AFM (ImAFM) with amplitude-dependent force spectroscopy (ADFS) [8–10]. Dynamic methods record local mechanical properties with a resolution in the range of the tip radius *R* of the AFM probe (typically 4 nm ≤ *R* ≤ 40 nm).

These methods are increasingly interesting for nanoscopic heterogeneous materials, such as nanocomposites. These materials are known to show nano-effects which improve the macroscopic properties of the composites beyond the rule of mixture. The mechanisms which cause the nano-effects are often described and hypothesized but seldom directly shown [11–14]. High-resolution measurements of the mechanical properties of nanocomposites give insights into these mechanisms, since they are able to separately measure material phases and interphases. Ideally, for a complete understanding of the underlying mechanism, a full structure–property relation is desired. A counterpart for the high-resolution physical properties (“how”) is needed to describe the local structure (“what”).

For that purpose, one major drawback of AFM force spectroscopy needs to be overcome. Despite the fact that AFM, in general, has a high sensitivity for physical properties and physical material contrasts, it is usually considered to lack sensitivity to detect chemical or structural information. Therefore, the magnitude and extent of different material phases and material interphases are usually deduced from AFM topography. This is problematic because subsurface structures are not taken into consideration even though they might be relevant for the physical properties [2,15]. Also, with increasing resolution and decreasing size of heterogeneous structures, AFM topography is often not conclusive.

To overcome these disadvantages, complementary measurements are performed. Methods such as SEM and EDX are able to image structural contrasts with a lateral resolution on the order of magnitude of the AFM tip size or higher [16–19]. However, since those are separate measurements one has to find again the same region of interest on the sample and lateral coordinates have to be synchronized with those of the force spectroscopy measurements. Although this can be automated, this operation is still an additional source of error. Complementary AFM measurements are easier to implement, since no additional sample preparation is necessary [20].

There is a number of AFM-based methods, such as tip-enhanced Raman spectroscopy (TERS) [21], AFM-based infrared spectroscopy (AFM-IR) [16,22], noncontact AFM (ncAFM ) [23–24], chemical AFM (cAFM) [25–26], and Kelvin probe force microscopy (KPM) [27] which were developed to identify local chemical or structural specificities in the samples. All the methods mentioned above are, to different degrees, advanced techniques which require additional equipment and expertise. TERS and AFM-IR are hybrid setups which include additional Raman and IR instrumentation, respectively. The effort to perform TERS or AFM-IR experiments is only justified if a detailed analysis of the chemical structure is needed. In this study, however, we aim to identify the material contrast which is provided by more accessible in situ methods. TERS and cAFM require a rather fragile modification of the AFM tip, which is usually not optimized for mechanical measurements. The methods that come closest to the requirements set in this study are KPM and ncAFM. KPM makes use of the interactions between the tip and the sample when an electric field is applied. Khorasani and coworkers identified nanoparticles (exposed and subsurface) in an epoxy/boehmite nanocomposite by measuring the surface potential by means of KPM [9]. A disadvantage of KPM is that, in addition to the apex of the tip, the sides of the tip are also interacting. This leads to a decrease in the lateral resolution compared to other AFM methods, such as tapping [28]. ncAFM is a more universal applicable method since it is carried out over the whole regime of attractive forces: It is sensitive to electrostatic forces (long range, >30 nm), van der Waals forces (intermediate range, <10 nm), and chemical forces (short range, <2 nm). By keeping the system in equilibrium (avoiding a snap onto the sample surface) the attractive forces at the surfaces are mapped, recording the change of resonance frequency due to the interacting attractive forces [23]. This method has not only achieved a lateral atomic resolution, but also enabled the identification of chemical structures down to single atoms. For these remarkable results, the sample has to be very smooth (ideally a crystal plane) and preferably free of an ambient water film (ultra-high vacuum AFM, UHV AFM). Again, these requirements cannot be met by most samples or most AFM setups. However, the principle idea that local attractive forces *F*_attr_ are specific for the local chemical composition and structure (as shown before by ncAFM [15,29] and NF-DAFM [30]) can also be utilized for force spectroscopy under ambient conditions.

In this study, we propose to use the information inherent to force spectroscopy to assess both the structure and properties of a given sample in one measurement. For that purpose, we evaluate *F*_attr_ acting between the AFM tip and the sample surface as an additional independent channel which can be used to identify the local chemical composition [31]. The attractive forces are acting on the AFM tip during the approach of the tip towards the sample surface (not to be confused with the adhesion force *F*_adh_ needed to separate the tip and the sample upon retraction). These attractive forces are highly specific for the chemical structure of a targeted sample volume [32–35]. This is an easily accessible parameter which does not require additional measurements or additional methods. The analysis of *F*_attr_ can also be put into practice for already obtained data and can be subsequently exploited as an additional characterization parameter.

## Methodology

**Figure 1:**
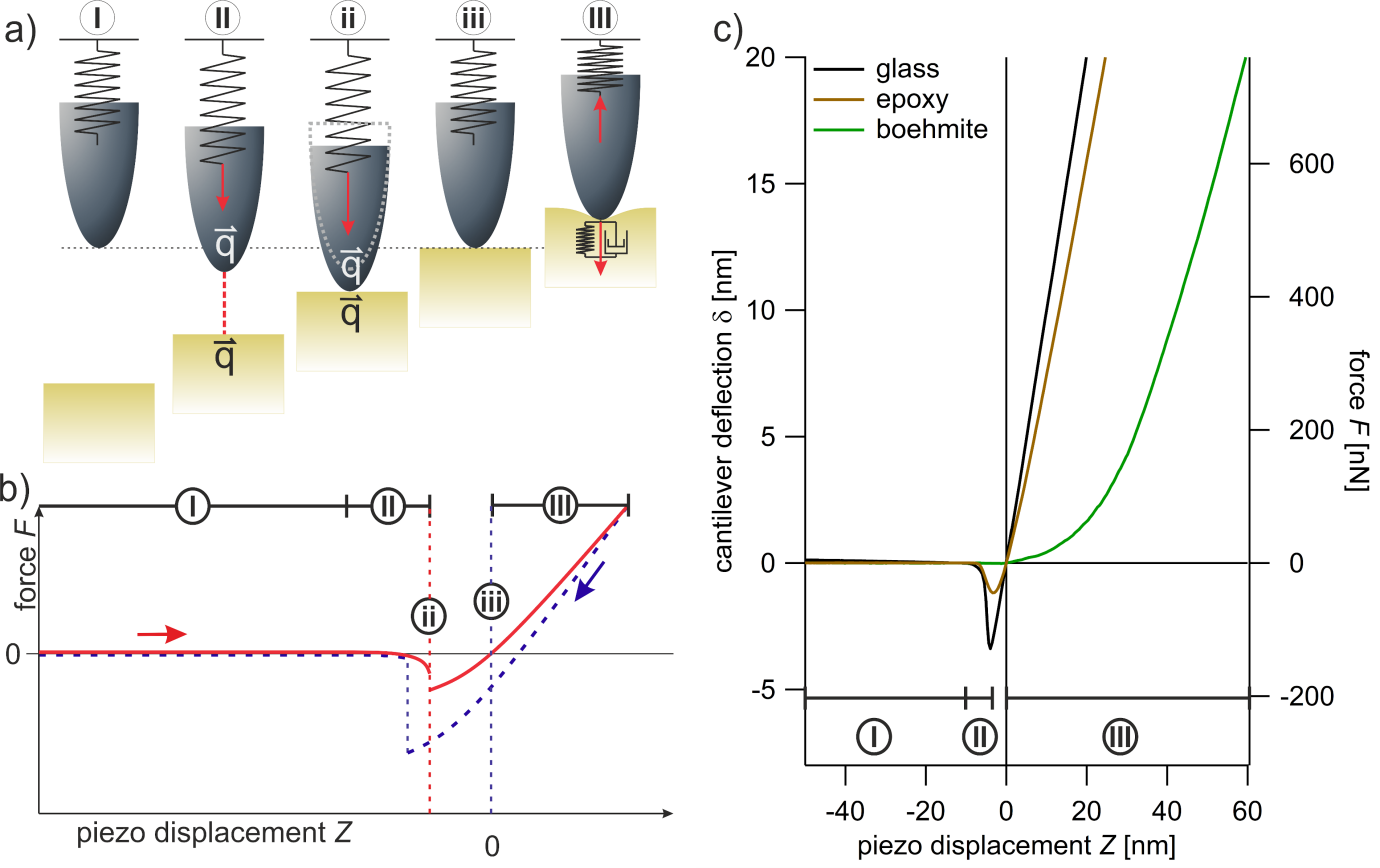
(a) Representation of tip–sample interactions. (b) Schematic drawing of a FDC. (c) FDCs (approach, averaged from at least 50 single curves) acquired from bulk materials: boehmite (green), epoxy (brown), and glass (black, for comparison). With (I) zero line, (II) regime of attractive forces, (ii) JTC, (iii) contact, and (III) contact line. The measurements were performed with the tip A.

For this method-focused study, AFM force–distance curves are used for proof of concept, since this method is well known as a reliable tool for measuring mechanical properties with a high lateral resolution [1–2,36–37]. FDC is not a scanning mode: The AFM probe, a paraboloid-shaped tip with a typical radius 4 nm < *R* < 40 nm is held at a defined *x*,*y* position while it approaches the sample surface by using a Z-piezo positioner. The tip is attached to a cantilever which can be described as an elastic spring following Hooke’s law:

[1] $$F=k_{\text{c}}\delta ,$$

with force *F*, spring constant of the cantilever *k*_c_, and cantilever deflection δ. In this way, the forces acting on the tip are measured by recording the deflection δ of the cantilever. While decreasing the distance between the tip and the sample, the cantilever deflects toward the sample (attractive forces *F*_attr_, −δ, decreasing the tip–sample distance, ζ) or away from the sample (repulsive forces *F*_rep_, δ, deforming the sample by *D*), depending on which interacting forces are dominant. The tip–sample distance, ζ, is given by:

[2] ζ(Z)=δ+D−Z,

with piezo displacement *Z*, deflection δ, and deformation *D*. This is explained in detail in Supporting Information File 1. For simplicity, all FDCs in this study are shown as δ (force *F*) as a function of *Z*, and *Z* is corrected for the point of contact in equilibrium (δ = 0), Figure 1 (iii), to be *Z* = 0.

Due to the sum of all interacting forces, a FDC shows three typical regimes upon approach, as depicted in Figure 1. Figure 1(I) zero line: when the tip and the sample are far away from each other. Interacting forces are not detectable and δ is zero which equals the free equilibrium position of the cantilever. Figure 1(II) regime of attractive forces: upon further approach of the sample and the tip, attractive forces start to govern and the cantilever is bent towards the sample. The attractive forces between the sample and the tip increase up to a point at which they exceed the gradient of *k*_c_. Figure 1(ii) jump to contact (JTC): a discontinuity where the system is not in equilibrium and the tip snaps onto the sample. Also at this point, the measurable maximum of the attractive forces is reached. Figure 1(iii) contact: attractive and repulsive forces are in balance and the cantilever reaches its equilibrium position again. Figure 1(III) contact line: upon further approach, the repulsive forces are dominant and the cantilever is pushed away from the sample. The deflection δ corresponds to the applied force, *F*_rep_, according to Equation 1. At a maximum deflection, δ_max_, (*F*_max_) the approach is stopped and the sample is withdrawn until the contact is lost at the jump-off-contact (JOC) and the zero line is reached again (only shown in Figure 1b, blue dashed line). In this study, only the approach part of the acquired curves is considered and evaluated. Hence, only attractive forces from the approach part are evaluated, which are not identical to the adhesion forces, dominated by the force needed to separate the tip and the sample after a forced contact.

We want to emphasize that at JTC, Figure 1(ii), the system is not in equilibrium and, therefore, one FDC cannot be considered as one continuous measurement, but rather a succession of measurements. In this study, the regime of attractive forces up to JTC, Figure 1(II) and the regime of repulsive forces from an applied force *F* = 0, Figure 1(III) are considered two independent measurements. This becomes comprehensible when taking the different origins of the acting forces into consideration. For that, we refer in the following paragraphs to the works of Israelachvili, Butt, and Parsegian [38–40], which we also recommend for further reading on the subject.

The attractive force acting between the tip and the sample is due to interacting dipoles and their generated fields when approaching one another. Only in a very close proximity between the tip and the sample, when the distance is in the range of molecular bonds and the orbitals can overlap, the chemical force comes into play. However, we consider the contribution of the chemical force negligible for experiments under ambient conditions as presented here. Also, we do not experience electrostatic interactions since the zero line, Figure 1(I), is stable up to distances of *Z* < −5 nm. Hence, as the main source of the measured attractive forces we consider electrodynamic interactions (caused by charge fluctuations in dipoles), commonly summarized under the term van der Waals forces. For the interaction of single neutral molecules separated by a distance *r*, the van der Waals force *F*_vdW_ includes three different types of dynamic dipole–dipole interactions: Keesom interaction (dipole–dipole), Debye interaction (dipole–induced dipole) and London interaction (transient dipoles). The van der Waals work *W*_vdW_ (force *F*_vdW_) needed to bring single neutral molecules from infinite to a finite separation *r* correlates with the inverse of the sixth power of the distance, −*C*/*r*^6^ (−6*C*/*r*^7^) and with the different positive coefficients *C*_Keesom_, *C*_Debye_, and *C*_London_. This is valid for the interaction of single molecules. In order to transfer this concept to bodies much larger than molecules, a further step is needed. This was carried out by Hamaker, who used a pairwise summation approximation to investigate the interactions between bodies, leading to the Hamaker coefficient *A*_Ham_ [41]. Consequently, the interaction between bodies with a distance ζ depends also on their geometry. In the case of AFM experiments, the geometry is assumed to be a sphere with radius *R* (tip) near a planar thick wall (sample). Usually, an AFM probe is characterized by the curvature of the apex of the tip which is in physical contact with the sample. For applying mechanical contact models, the geometry of the curvature is described as a paraboloid or a sphere with a tip radius *R*. However, this does not represent the actual geometry of the probes which have an electrodynamic interaction. Fronczak and coworkers found that the portion of the tip that contributes to the van der Waals forces is indeed much larger than that interacting mechanical forces and can be described by the effective radius *R*_eff_. *R*_eff_ is deduced by the calibration of the system with a tip–sample pairing with known Hamaker constants [35]. With *R*_eff_ and ζ << *R*_eff_, *W*_vdW_ and *F*_vdW_ can be described by:

[3] $$W_{\text{vdW}}=-\frac{A_{\text{Ham}}}{6}\frac{R_{\text{eff}}}{\zeta }\text{ and}$$

[4] $$F_{\text{vdW}}=\frac{-\text{d}W_{\text{vdW}}}{\text{d}\zeta }=-\frac{A_{\text{Ham}}}{6}\frac{R_{\text{eff}}}{\zeta^{2}}\text{, respectively}\text{,}$$

with the consequence that, instead of a decrease with the inverse of the seventh power of the distance (1/*r*^7^) as predicted for single molecules, *F*_vdW_ for larger bodies decreases with an increase in distance (1/ζ^2^). Since *R*_eff_ > *R*, this distance dependency makes *F*_vdW_, in general, distinguishable from short-ranged chemical forces and long-ranged electrostatic forces and also dominant in an AFM setup under ambient conditions.

But how is this property specific for different materials? In simplified terms, oscillating dipoles emit electromagnetic waves which in turn generate oscillating dipoles in an adjacent body. The response of a specific material in an oscillating electromagnetic field *E*(*f*) is described by the dielectric constant ε_r_(*f*) which can be extracted from the absorption spectrum of the material. Hence, how materials (e.g., the sample (S) and the tip (T)) interact in a medium (m) is defined by the difference in the dielectric responses. The Hamaker coefficient *A*_Ham_ can be derived from the relative differences of the dielectric constant ε_S_(*f*), ε_T_(*f*), and ε_m_(*f*), summed up over all the frequencies at which the fluctuations can occur (UV–vis–IR), according to:

[5] $$A_{\text{Ham}}\left(\zeta \right)=\frac{3kT}{2}\sum_{f_{k=1}}^{f_{n}}\left(\frac{\varepsilon_{\text{S}}-\varepsilon_{\text{m}}}{\varepsilon_{\text{S}}+\varepsilon_{\text{m}}}\right)\left(\frac{\varepsilon_{\text{T}}-\varepsilon_{\text{m}}}{\varepsilon_{\text{T}}+\varepsilon_{\text{m}}}\right)\text{Rel}\left(\zeta \right).$$

Bodies made from the same material show the highest attraction to each other, since the emitting field and the absorption by the oscillating dipoles are in resonance. Dissimilar materials attract each other less, which might even lead to repulsion. The attraction between two dissimilar materials in a defined medium is specific for that constellation. In our case, the material and the shape of the tip (ε_T_, *R*_eff_) are always the same for the entire duration of the measurement. The same is true for the medium (ε_m_). Any change in the measured attractive force *F*(ζ) (≈*F*_vdW_) is due to the local absorption spectrum of the sample ε_S_ and, therefore, to a change in *A*_Ham_.

Das and coworkers showed that instead of fitting the force plot *F*(ζ) (as shown in Supporting Information File 1) the deflection distance δ_JTC_ (= *F*_attr_/*k*_c_) can be used to estimate *A*_Ham_ [33]:

[6] $$A_{\text{Ham}}=\frac{4}{27}\frac{6k_{\text{c}}\delta_{\text{JTC}}^{3}}{R_{\text{eff}}}.$$

**Figure 2:**
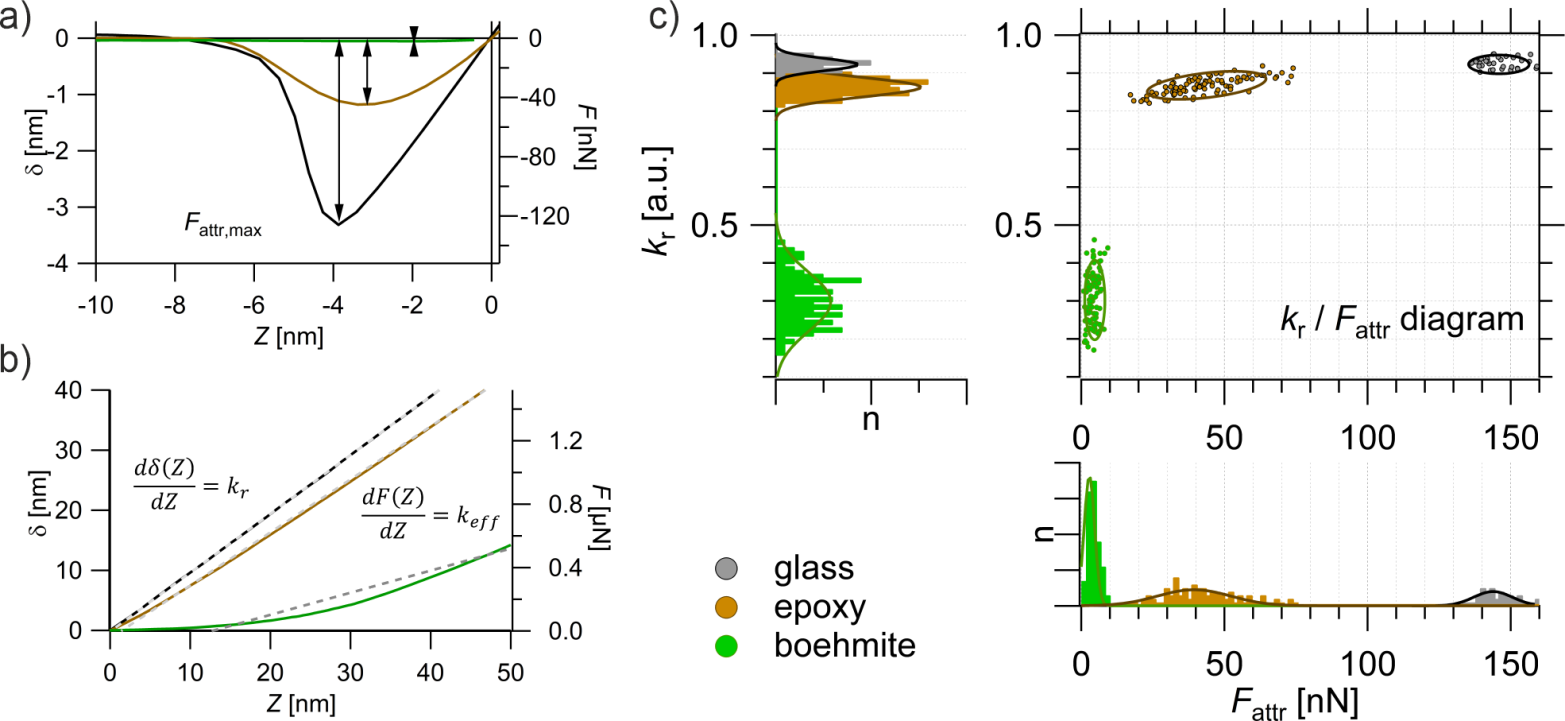
FDCs (averaged from at least 50 single curves) of bulk materials: boehmite (green), epoxy (brown), and glass (grey). (a) Regime of attractive forces with *F*_attr_. (b) Regime of repulsive forces with *k*_r_ and *k*_eff_. (c) Property domain: top left and bottom right are histograms of *k*_r_ and *F*_attr_, respectively, of single FDC; top right: *k*_eff_/*F*_attr_ diagram for the structure–property correlation. The measurements were performed with the tip A.

This is a valid approach for estimating the absolute value of *A*_Ham_ when *k*_c_ is very small and JTC occurs early, before an additional deflection occurs. In the present work, cantilevers with high spring constant values are used and the effective radius *R*_eff_ is unknown. Therefore, the absolute value for *A*_Ham_ cannot be estimated. However, the maximum attractive forces are clearly dominated by the van der Waals interactions and correlate with *A*_Ham_. As an example, the specificity and sensitivity of *F*_attr_ for different materials is shown in Figure 2a for averaged example curves (average of approximately 50 curves) of different neat solids: glass (black), epoxy (brown), and boehmite (green).

In contrast, repulsive forces acting between the tip and the sample upon contact (Figure 1(iii) and Figure 2b) are due to the exchange interaction, also called Pauli repulsion. This is a quantum mechanical effect that appears when identical particles are forced to occupy the same region of space and, hence, the Pauli exclusion principle is violated. Upon further approach (Figure 1(III)), the tip and the sample are deformed due to the applied force *F* according to their mechanical properties. For reasons that are explained below, we assume that the local mechanical properties of the sample can be represented by an elastic spring with a spring constant *k*, although this is certainly a simplification. With a cantilever deflection δ, a cantilever spring constant *k*_c_, the sample spring constant *k*, and a tip–sample distance *Z*, the elastic response of the whole setup can be described by

[7] $$\delta =\frac{k}{k_{\text{c}}+k}Z=k_{\text{r}}Z,$$

yielding a dimensionless relative spring constant (or stiffness) *k*_r_, with *k*_r_ ≤ 1. A detailed derivation of *k*_r_ and a discussion regarding this parameter can be found in Supporting Information File 1. For a sample that is not measurably deformed by the applied force (*k* >> *k*_c_), *k*_r_ ≈ 1. This is the case when the sample is stiffer than the experimental setup, which marks the limitation of the method. Compliant or soft samples yield a stiffness value *k*_r_ < 1; the lower the value the softer/more compliant the sample. The evaluation of this parameter is shown in Figure 2b, again for averaged example curves (average of ≈50 curves) of glass (black), epoxy (brown), and boehmite (green). As seen from this plot, *k*_r_ is the slope of a linear fit of δ(*Z*) = *k*_r_*Z*. This value does not differentiate between plastic (soft) or elastic deformations (compliant), it actually reflects the overall characteristics of the contact part of an FDC. Being able to describe the mechanical properties with a single parameter might be a simplification; however, this is essential for the subsequent statistical data analysis, which will provide much more insight.

In summary, each single FDC yields two parameters, the maximum attractive force *F*_attr_ and the stiffness *k*_r_. These results are shown and treated in two different ways. First, since FDCs are measured in an *x*,*y* grid, spatially resolved maps of *F*_attr_ and *k*_r_ can be obtained. They will be referred to as the spatial domain. Second, the parameters *F*_attr_ and *k*_r_, can be treated statistically. This is done by plotting them as histograms, individually or correlated as *k*_r_(*x*_i_,*y*_i_) as a function of *F*_attr_(*x*_i_,*y*_i_), which leads to the *k*_r_/*F*_attr_ diagram, as shown in Figure 2c. By plotting the results in the *k*_r_/*F*_attr_ diagram, the information about the spatial distribution is lost. However, insight into the structure–property correlation that governs the behavior of the sample is gained. This will be referred to as the property domain.

## Results and Discussion

### Bulk material

Neat bulk materials, which will be later used in composites, are measured separately to establish the capability of the proposed method. Measurements of bulk epoxy, bulk boehmite, and glass (as a reference) were already introduced in Figure 1 and Figure 2 in order to illustrate the method. Approximately 50 FDCs per material were evaluated for their parameter sets *k*_r_ and *F*_attr_, as shown in Figure 2a and Figure 2b. Figure 2c shows the property domain of the measurements. On the top left, the histogram of the stiffness *k*_r_, acquired from glass (grey bars), neat epoxy (yellow bars), and boehmite (green bars) is shown. Boehmite shows a wide range of *k*_r_ values (0.2 < *k*_r_ < 0.4), which is due to its anisotropic ductility caused by its slip planes [39]. As expected, glass is the stiffest material and shows values of *k*_r_ ≈ 0.95. Epoxy is softer/more compliant than glass with *k*_r_ ≈ 0.85, overlapping with the mechanical characteristics of glass. Hence, the mechanical properties of epoxy and glass are similar, and a mechanical measurement cannot be used to distinguish between these two material phases. For that purpose, additional information is needed, which is provided by *F*_attr_.

At the bottom-right panel of Figure 2c, the histogram of *F*_attr_ is shown. The values obtained from glass (grey bars), neat epoxy (brown bars), and boehmite (green bars) are unambiguously distinguishable, since they show distinct distributions which do not overlap. The correlation of both parameters, the *k*_r_/*F*_attr_ diagram (the top-right panel of Figure 2c) shows a very distinctive pattern of accumulation points for the different materials. This method is valid for the measurements that were performed with a single AFM probe. Under this condition, any given FDC can be classified in the *k*_r_/*F*_attr_ diagram. This treatment can, therefore, be considered a manually performed principle component analysis (mPCA).

**Figure 3:**
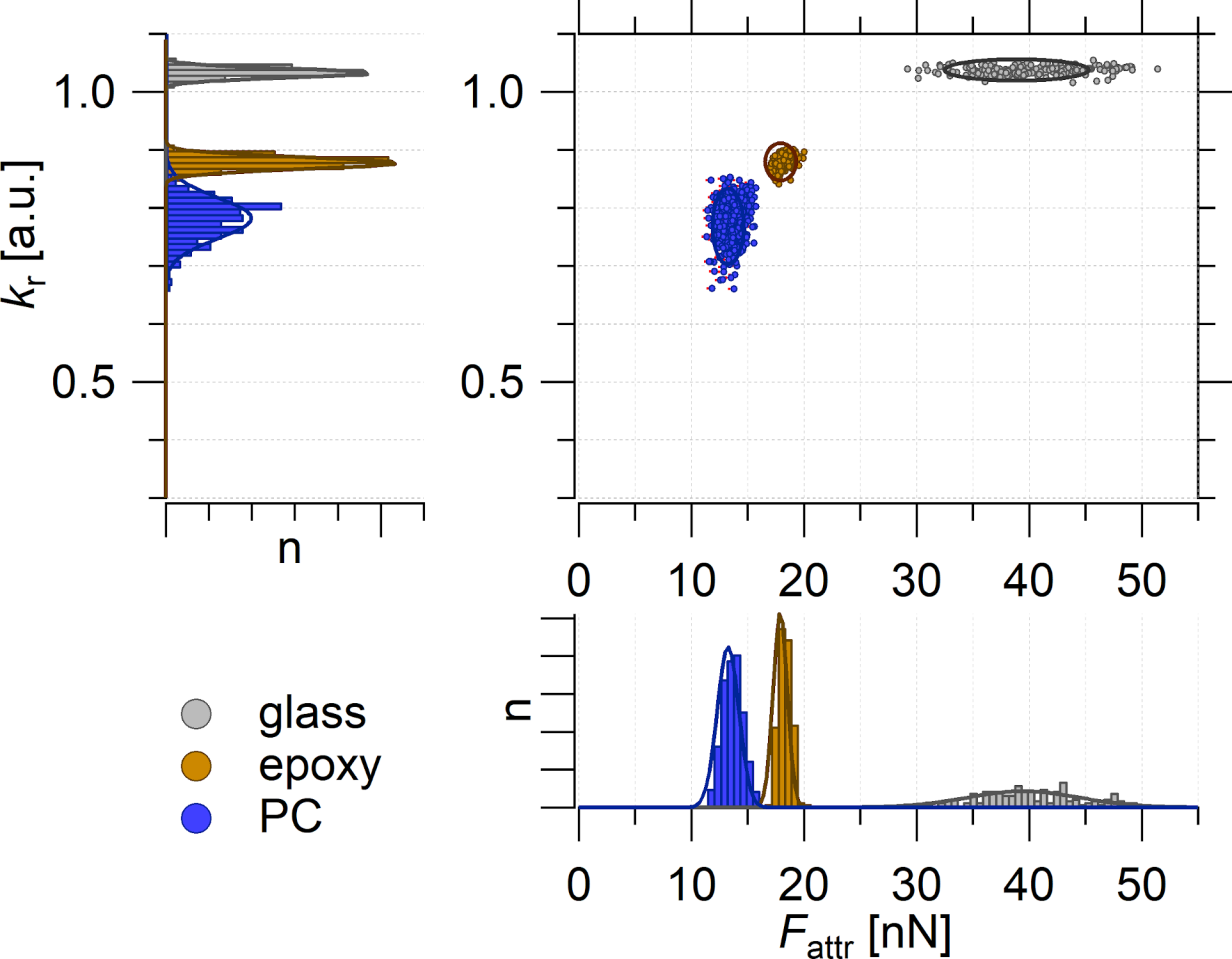
The property domain of FDCs (average of ≈100 single curves) of bulk materials: PC (blue), epoxy (brown), and glass (grey). Top-left and bottom-right panels are histograms of *k*_r_ and *F*_attr_, respectively. The top-right panel is the *k*_eff_/*F*_attr_ diagram for the structure–property correlation. The measurements were performed with the tip B.

Figure 3 shows the analogue results for bulk epoxy (brown) and polycarbonate (PC, blue) and the reference measurement of glass (grey) in the property domain. When comparing the histograms of *k*_r_ with the histograms of *F*_attr_ it again becomes apparent that the distributions of values for PC and epoxy overlap in the case of *k*_r_, whereas, in the case of *F*_attr_, a clear distinction can be made. Note that the measurements shown in Figure 3 were performed with a different AFM probe (tip B) than the measurements shown in Figure 2 (tip A). Hence, the different scales for the *F*_attr_ values.

### Model sample epoxy/polycarbonate

**Figure 4:**
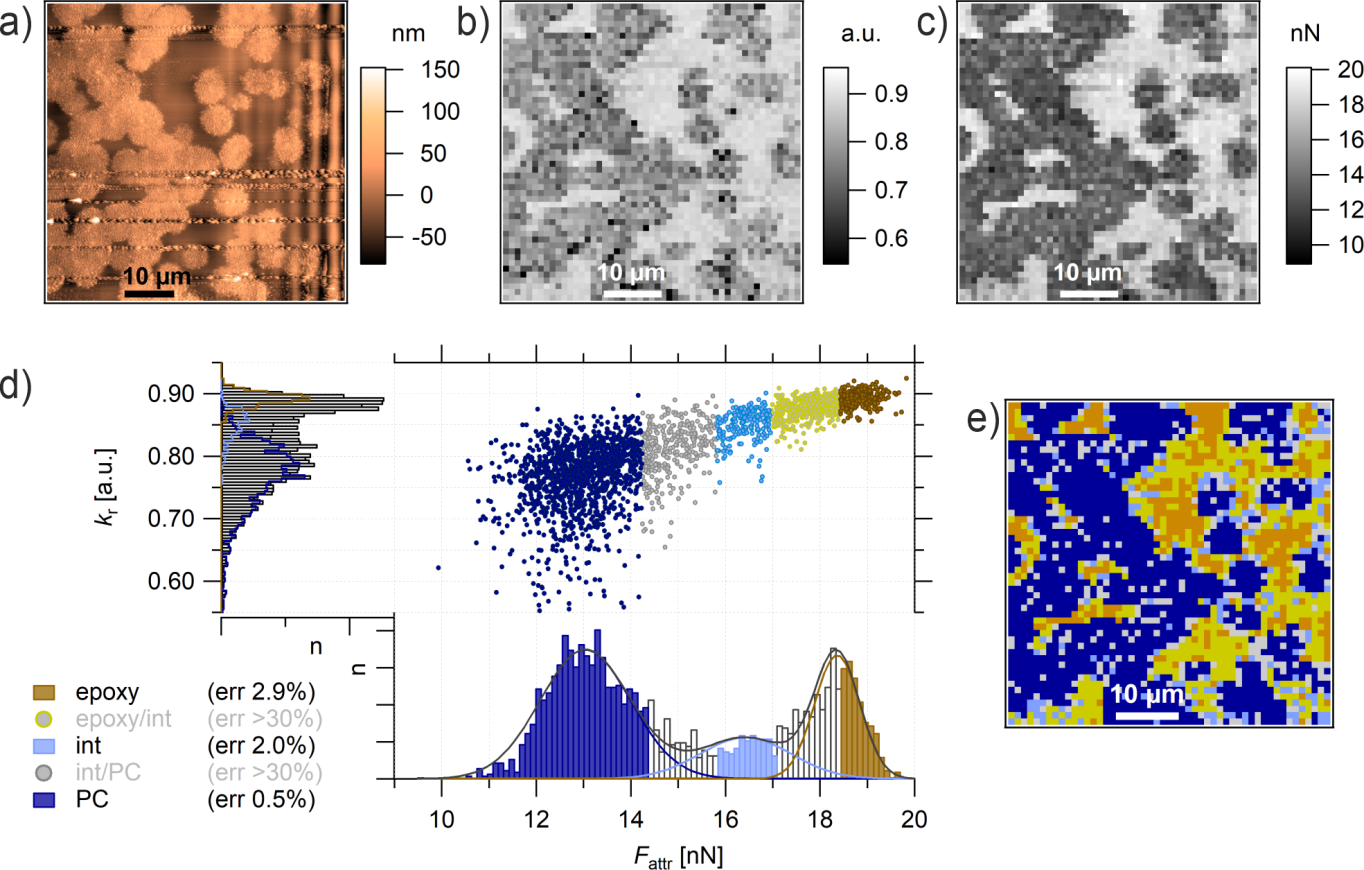
(a) AFM tapping-mode topography. PC and epoxy phases can be distinguished by the height difference. (b) *k*_r_ and (c) *F*_attr_ AFM FDC maps (60 × 60 points). (d) Property domain of the AFM FDC measurement with the *k*_r_ histogram (top left), *F*_attr_ histogram (bottom right) and *k*_r_/*F*_attr_ diagram (top right). The *k*_r_ histogram is deconvoluted with three Gaussian fits assigning the chemical structures to epoxy (brown) and PC (blue) and an intermediate behavior (int, light blue) to the measuring points (error <3%). Measuring points which a larger error (>30%) are found between the epoxy and int (grey yellow) or between int and PC (grey) (see also the *k*_r_/*F*_attr_ diagram, top-right panel). (e) mPCA: results of the property domain are shown in the spatial domain. The measurements were performed with the tip B.

Here, we consider a sample that was previously described in detail but analyzed in a different context by Cano Murillo and coworkers [19]. The sample is a binary composite consisting of PC and epoxy. At the beginning of the curing process, the two main components of epoxy, resin and hardener, have not yet reacted and the low molecular weight resin dissolves PC at elevated temperatures. During curing, when the crosslinking of resin/hardener occurs, the epoxy gradually increases its molecular weight and PC is precipitated into a spherulite structure. This structure makes the PC phase easily distinguishable from the epoxy phase in the spatial domain (Figure 4a–c).

The goal of this analysis is to establish the proposed method as an independent source of information about the chemical structure of the composites. The topography is used as a confirmation.

Comparing the topography (Figure 4a) with the *k*_r_ map (Figure 4b) and the *F*_attr_ map (Figure 4c), the maps clearly correlate with the features shown in the topography. Please note that the horizontal and the vertical lines shown in the topography are artefacts from the sample preparation. Those spots were excluded from the maps (Figure 4b and Figure 4c). A detailed description of the data treatment and the correlation between topography and *F*_attr_ values are provided in Supporting Information File 1 (Figure S1).

Beyond the similarities between the maps, we propose a statistical approach. For that we give up the spatial information (i.e., the spatial domain) and look at the distributions of the parameters in the form of histograms (i.e., the property domain) as shown in Figure 4d. On the left side, the bimodal distribution of the stiffness is shown (grey bars), which reflects the bimodal composition of the material. Any distinguishable mechanical response from a possible third phase (an interphase) cannot be distinguished, which was already reported by Cano Murillo and coworkers [19].

The histogram of *F*_attr_ at the bottom right of Figure 4d also shows two main peaks; however, an additional minor third peak emerges in between the main peaks. A deconvolution of the *F*_attr_ histogram with Gaussian distributions (envelope function: black line) shows that the normal distribution of *F*_attr_(PC) (blue line) and *F*_attr_(epoxy) (brown line) do not overlap. Due to this gap, a third intermediate peak *F*_attr_(int) (light blue line) can be unambiguously distinguished. However, the three distributions partially overlap and, therefore, only a limited range of *F*_attr_ values can be assigned to a material phase with an acceptable error (difference between an individual fit and the envelope function <3%).

The *k*_r_/*F*_attr_ diagram (Figure 4d, top right) provides a complete structure–property correlation. This becomes even more evident when transferring the findings of the deconvolution of the *F*_attr_ histogram to the *k*_r_/*F*_attr_ diagram: epoxy (brown markers), int (light blue markers), PC (blue markers). As mentioned above, in this study we are only interested in the specificity of the parameter *F*_attr_ and, therefore, we accept the rather abrupt vertical divisions in the *k*_r_/*F*_attr_ diagram. By incorporating an additional deconvolution of the *k*_r_ histogram this can be improved.

In order to show these results in the spatial domain as a map (Figure 4e), the measuring points where *F*_attr_ did not provide unambiguous structural assignment are labeled in grey (PC or int) and in yellow (int or epoxy). The structural map shown in Figure 4e is, again, in very good agreement with the topography data and identifies the constituents of the composite epoxy (brown) and PC (blue) very well. The measuring points identified as intermediate species (light blue) are situated at the phase boundaries. As mentioned before, at this resolution we do not expect a measurable interphase which has distinguishable properties. The reason for the intermediate behavior is more likely the presence of both phases in the measured volume and, therefore, a mixed behavior. This is supported by the corresponding mixed mechanical behavior, as seen in the *k*_r_/*F*_attr_ diagram (Figure 4d, top right). It has to be taken into account that the topography only shows a cross section of a three-dimensional structured composite and PC spherulites might extend underneath the epoxy and vice versa. In the case that a PC spherulite is only shielded by a sufficiently thin epoxy layer, it probably contributes to the measured properties without being visible in the topography.

The important results of this experiment are that *F*_attr_ 1) is suitable parameter for distinguishing two different organic compounds (PC and epoxy), 2) is more sensitive for identifying material differences than the mechanical properties, and 3) appears, in this case, to be even sensitive to subsurface structures.

### Model sample epoxy/boehmite

**Figure 5:**
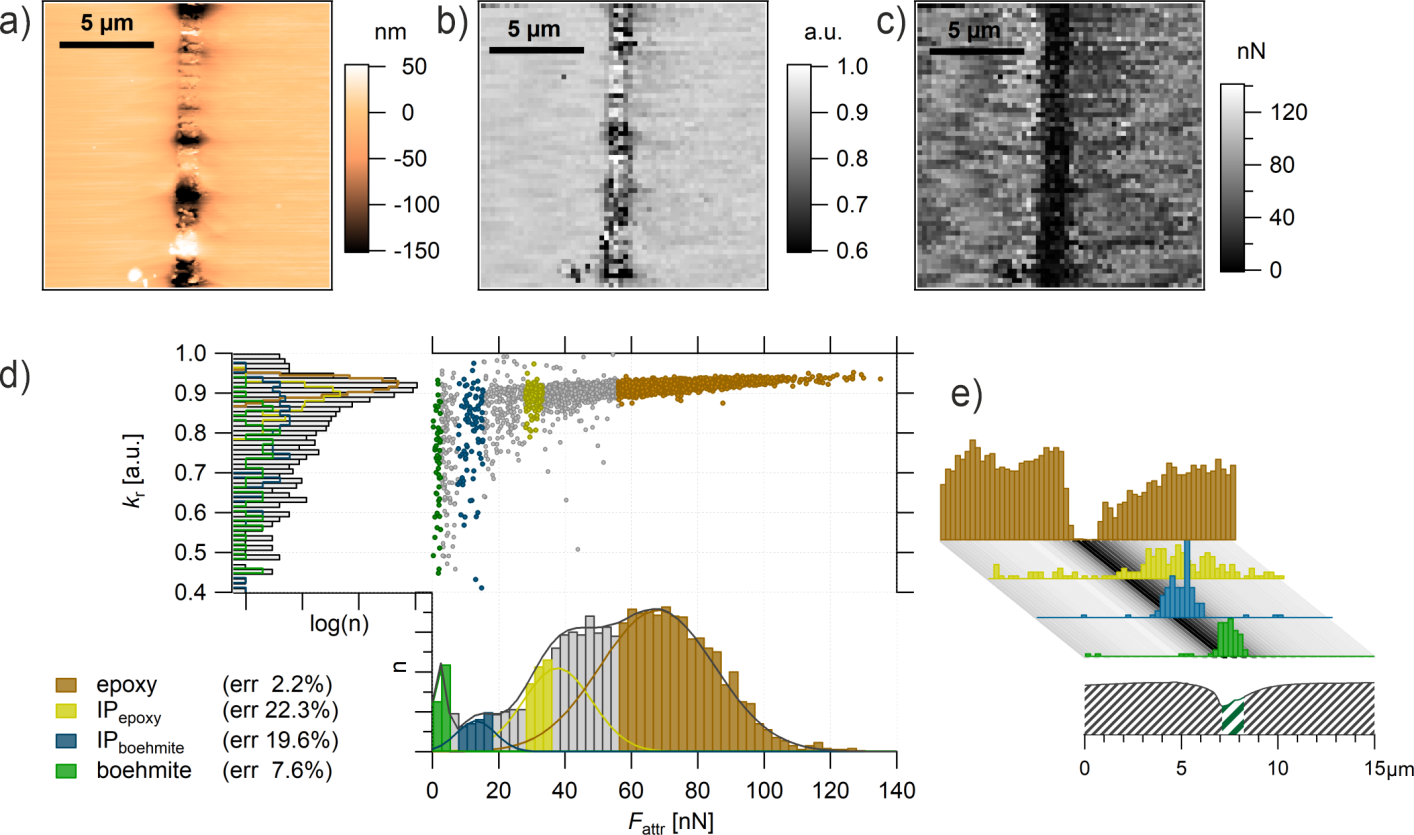
(a) AFM tapping-mode topography. Epoxy and boehmite phases can be distinguished by features that varied in height. (b) *k*_r_ and (c) *F*_attr_ AFM FDC maps (80 × 80 points). (d) Property domain of AFM FDC with *k*_r_ histogram (top left), *F*_attr_ histogram (bottom right), and *k*_r_/*F*_attr_ diagram (top right). The *k*_r_ histogram is deconvoluted with four Gaussian fits assigning the chemical structures epoxy (brown), IP_epoxy_ (yellow), IP_boehmite_ (turquoise), and boehmite (green) to individual measuring points (see also the *k*_r_/*F*_attr_ diagram). (e) Assignable measuring points in each column were summed up, leading to a histogram of each species as a function of the distance to the boehmite structure (topography profile at the bottom). The measurements were performed with the tip C.

The second model sample consists of an organic phase and an inorganic phase and is described in detail by Khorasani and coworkers [16]. A layer of boehmite is sandwiched between epoxy layers, as seen in Figure 5a–c which shows the cross section of the three layers. The layers were built from left to right, which means that the left-side epoxy was cured before acting as a substrate for the boehmite layer. The right-side epoxy, however, was cured in the presence of boehmite. This led to an interphase region in the right-side epoxy, which differs significantly in its properties and structure when compared to the neat bulk epoxy. This is discussed in detail in [16]. Analogous to the first model sample, the material phases can be clearly identified by the topography shown in Figure 5a.

Comparing the topography with the *F*_attr_ map (Figure 5a and Figure 5c, respectively) the specificity of *F*_attr_ for boehmite and epoxy is indicated. However, by comparing with the stiffness map in Figure 5b, it can be seen that the mechanical properties are not specific. Especially inside the boehmite layer, a broad range of stiffness values are measured. This is confirmed by looking at the property domain shown in Figure 5d. The stiffness histogram shows a rather broad and not differentiable peak, and the characteristic values for different components cannot be established. In the *F*_attr_ histogram, at low *F*_attr_ values, a group of points (green) is clearly distinguishable, showing an undisturbed peak. In the spatial domain, shown in Figure 5e, the position of these points (green) coincide with the boehmite layer. Therefore, it can be assumed that these *F*_attr_ values (<7 nN) are typical for boehmite. Also, one broader peak at high *F*_attr_ values can be assigned to epoxy (brown).

However, the specificity of *F*_attr_ for the epoxy interphase region IP_epoxy_ (yellow) is more complex. Here, epoxy shows slightly lower *k*_r_ values and significant lower *F*_attr_ values. This is not a bimodal distribution, but rather a gradual effect as seen in the spatial domain of *F*_attr_, shown in Figure 5e (decrease of epoxy signal, brown and increase of IP_epoxy_, yellow).

In order to confirm a structural or a chemical change, a complementary AFM-IR method was used. This hybrid setup is comprised of an AFM and a tunable pulsed laser source focused on the sample volume underneath the AFM tip. The absorption at distinct wavelengths is measured by detecting the thermal expansion of the material by the means of the amplitude of the tip (IR amplitude) [22].

**Figure 6:**
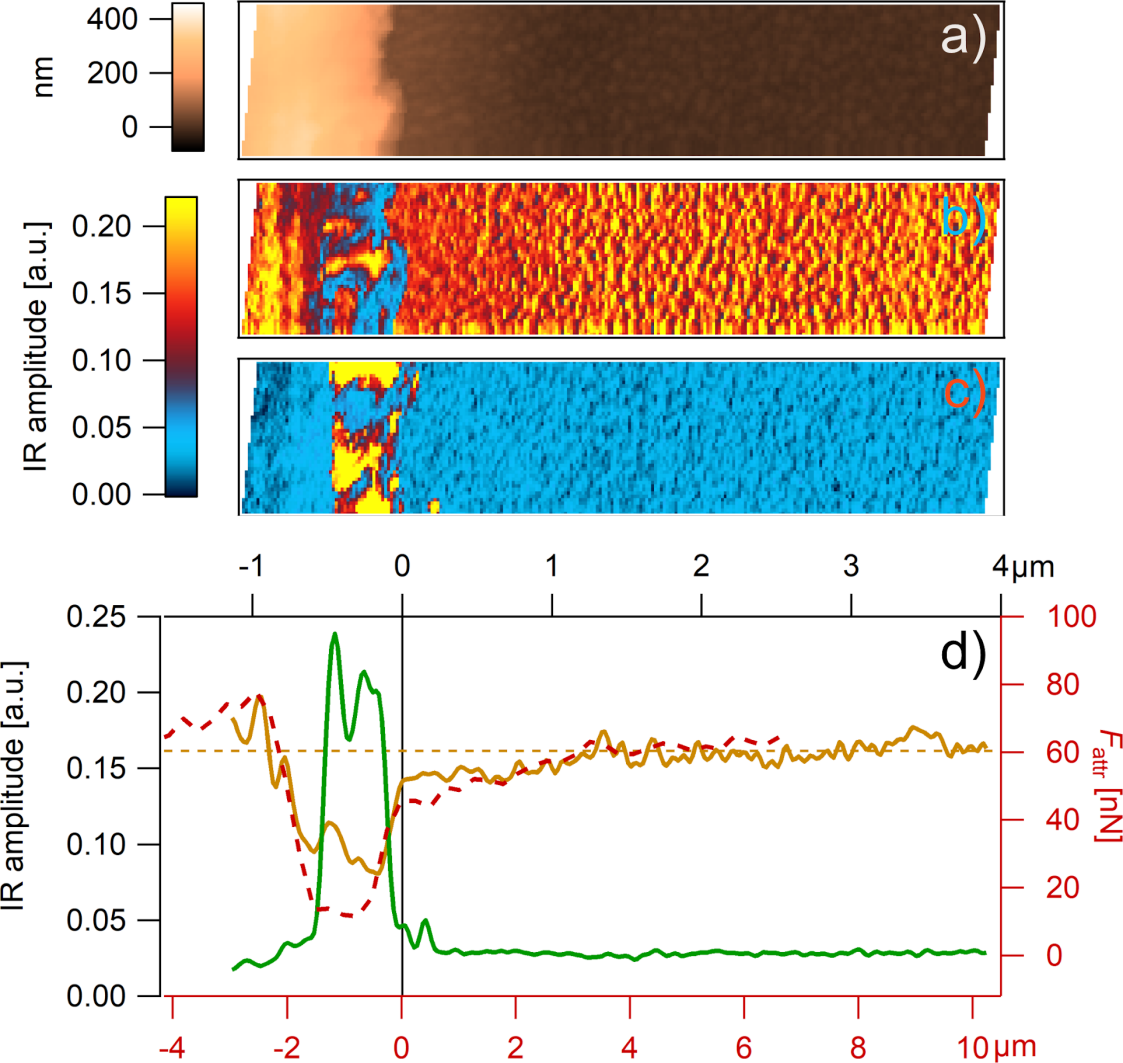
AFM-IR measurements. (a) AFM height, (b) IR amplitude at 1512 cm^−1^, and (c) IR amplitude at 1070 cm^−1^. (d) Average of the IR amplitude at 1512 cm^−1^ (brown line) and at 1070 cm^−1^ (green line). For comparison, averaged *F*_attr_ values are shown (red dashed line) as a function of *x* (distance to the boehmite structure). The AFM-IR measurements were performed with the tip D.

For this measurement, the sample (topography shown in Figure 6a) was scanned while the IR amplitude of the material was measured at one specific wavelength for epoxy (1512 cm^−1^, Figure 6b) and at another for boehmite (1070 cm^−1^, Figure 6c). In Figure 6d, the averaged IR amplitude, measured at 1512 cm^−1^ for epoxy (brown) and at 1070 cm^−1^ for boehmite (green) is shown as a function of the distance to the boehmite layer. Again, the most striking feature is the boehmite structure. However, the gradual decrease in the IR amplitude(*x*) in the epoxy interphase region is also clearly visible and in very good agreement with *F*_attr_(*x*), which is also shown in Figure 6d (red dashed line).

The decrease in the IR amplitude may have two possible reasons: either the absorption band is shifted or the intensity of the absorption band is decreased. Although a shift of the bands due to hydrogen bonding is possible, it is very unlikely that a shift of ±10–30 cm^−1^ in the absorption band has a measurable effect on the attractive forces. We hypothesize that the density of the bonds/dipoles decreases, leading to a decrease in both signals, IR amplitude, and *F*_attr_. The epoxy network affected by the presence of boehmite was already shown to have a different structure, which might lead to a decreased density, but not to a significantly altered stiffness [42–43]. This is confirmed by the *k*_r_/*F*_attr_ diagram (Figure 5d) which shows only a slight change in *k*_r_.

This hypothesis is also supported by the left-sided epoxy measurements. The left-sided epoxy was initially cured facing air which leads to a denser structure at the surface, usually referred to as the skin effect [44]. In both measurements, the IR amplitude and *F*_attr_ show significant higher values for the skin region. Again, this is not necessarily reflected in the stiffness below the glass transition. We, therefore, conclude that the change in *F*_attr_ does not directly reflect a chemical effect, but rather a structural one.

Both measurements also show evidence of a boehmite/epoxy composite inside the boehmite layer, IP_boehmite_. The IR amplitude for the epoxy decreases significantly inside the boehmite structure, but not to zero (Figure 6d, brown line). This is in very good agreement with the *F*_attr_ values between 7 and 20 nN in the property domain (Figure 5d, IP_boehmite_: turquoise) which are exclusively found inside the boehmite structure as seen in the spatial domain of Figure 5e (turquoise). The IP_boehmite_ phase shows a mixed behavior of epoxy and boehmite, and boehmite is the dominant influence.

To summarize the results of the second model sample: 1) boehmite and epoxy can be very well distinguished, 2) mixed and gradient behavior of boehmite/epoxy interphases can be quantified, and 3) structural changes of epoxy can be quantified by the parameter *F*_attr_.

### Epoxy/polycarbonate/boehmite composite

**Figure 7:**
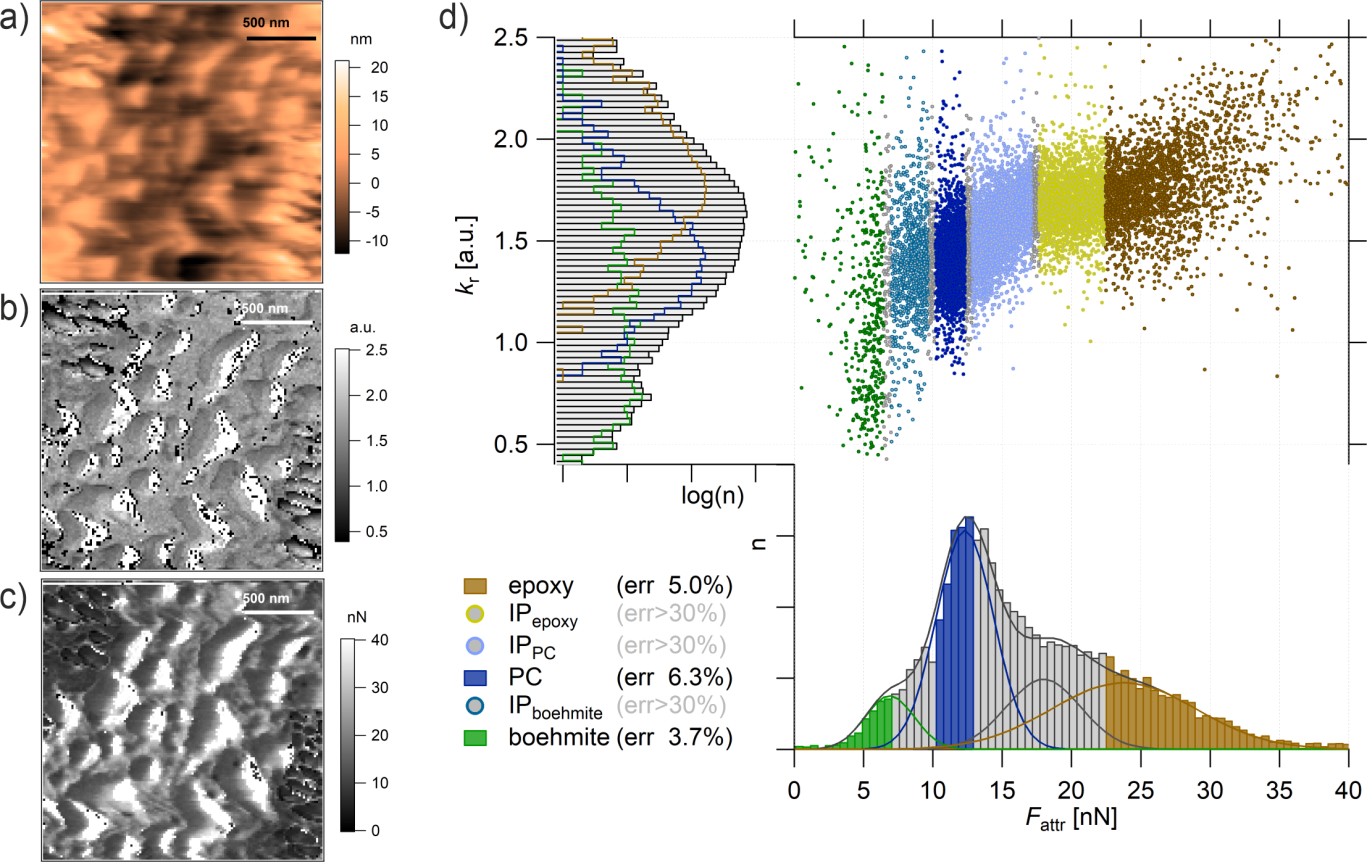
ImAFM ADFS (a) height, (b) *k*_r_, and (c) *F*_attr_ map (128 × 128 points). (d) Property domain of the *k*_r_ histogram, *F*_attr_ histogram, and *k*_r_/*F*_attr_ diagram. The *k*_r_ histogram is deconvoluted with four Gaussian fits assigning epoxy (brown), PC (blue), and boehmite (green) to the measuring points (error < 7%). The measuring points with a larger error of assignment (>30%) are IP_epoxy_ (yellow), IP_PC_ (light blue, between epoxy and PC), and IP_boehmite_ (turquoise, between PC and boehmite). See also the *k*_r_/*F*_attr_ diagram (top right). The measurements were performed with tip E.

The third sample is comprised of three materials: epoxy, PC and boehmite nanoparticles (NPs). Boehmite NPs, with a primary particle size of 20 nm, are dispersed and incorporated in electrospun fibers of PC with a diameter 1 µm < Ø < 10 µm (Supporting Information File 1, Figure S2). The electrospun fibers are subsequently embedded into epoxy. A detailed description of the sample preparation is found in the experimental section. As mentioned above, the unreacted epoxy is able to dissolve PC. In this case, the goal is to dissolve PC to a degree that boehmite NPs are released into the epoxy, which can be achieved with the right temperature treatment. A prolonged and elevated temperature can cause two opposite effects on the dissolving process: increase the solubility of PC and decrease the solubility of epoxy by increasing its molecular weight. To find the optimized temperature treatment it is crucial to be able to test the boehmite NPs interphase. Considering the small sample volume and the resolution needed for this task, a high-resolution method of AFM force spectroscopy was used, which is called intermodulation AFM. This dynamic method is able to scan the sample with a resolution comparable to the tapping-mode images, producing the equivalent of a FDC at each pixel. A 2 × 2 µm topography scan of the ternary system with 128 × 128 points is shown in Figure 7a.

**Figure 8:**
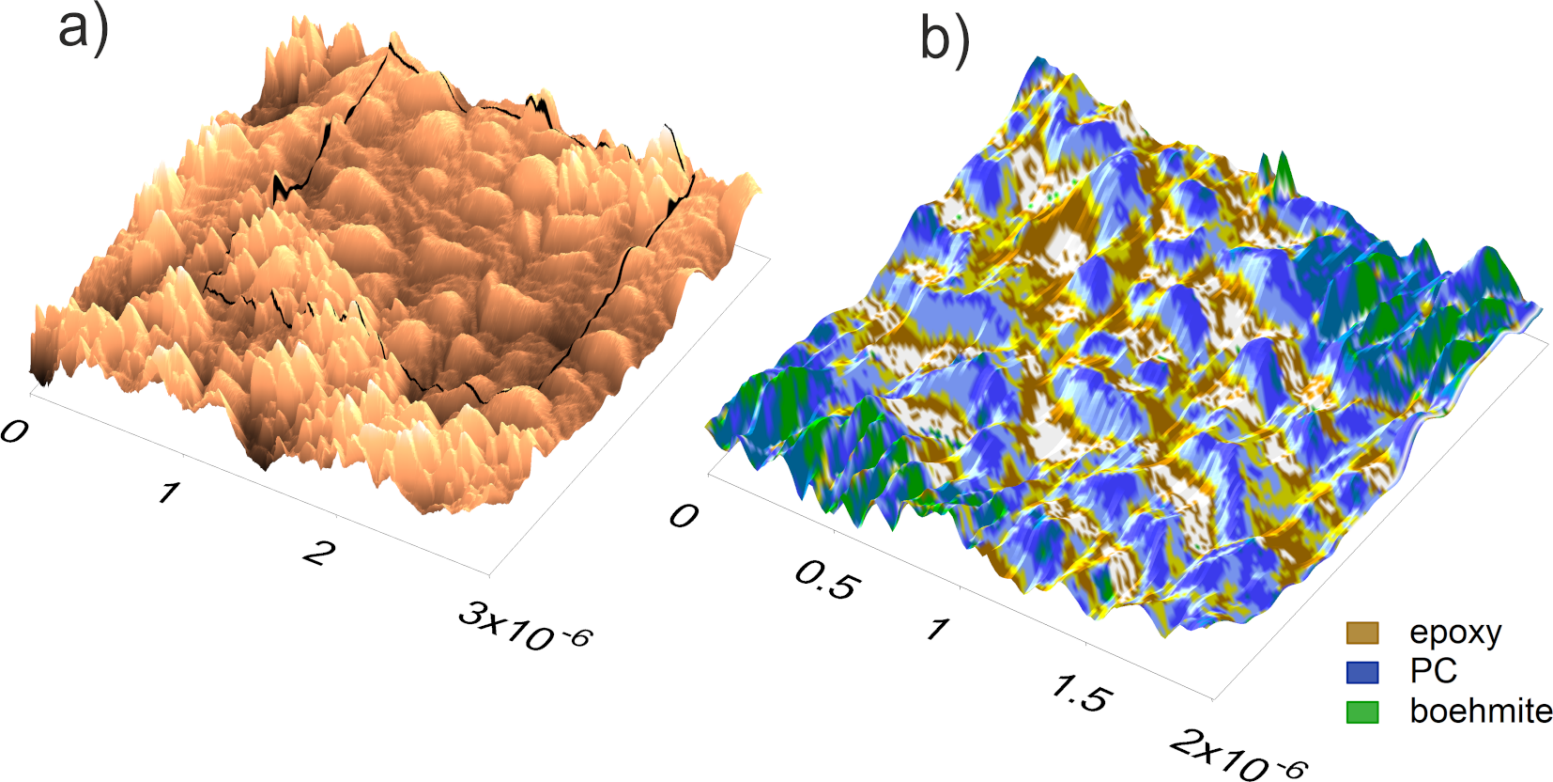
(a) AFM tapping topography, the black line indicates the position of (b). (b) mPCA via ImAFM ADFS measurements and *F*_attr_ deconvolution (spatial domain). PC (blue) is dissolved by epoxy (brown) but boehmite NPs (green) are not released into the epoxy matrix. The measurements were performed with the tip E.

Note that the topography is recorded as a by-product of the mechanical measurements by means of ImAFM ADFS. The measuring system is optimized for the mechanical measurement. For a better quality topography image, please see Figure 8a.

The topography shows three distinguishable areas, two rougher ones divided by a smoother one. However, it is not possible to assign the topography features to specific components. The stiffness map in Figure 7b gives few additional clues besides the existence of a compliant interphase present in the rougher structures (black points, *k*_r_ < 0.08). White points in Figure 7b and Figure 7c refer to unevaluable measuring points due to topography effects.

On the other hand, the *F*_attr_ map shown in Figure 7c gives a much improved contrast, which is also visible in the property domain of Figure 7d. In contrast to the *k*_r_ histogram, which shows one very broad peak, the *F*_attr_ histogram can be deconvoluted with four Gaussian fits: one is a rather narrow peak in the center (blue) assigned to PC, another one, also with a narrow shoulder at smaller values (green), is assigned to boehmite NPs. The highest values of *F*_attr_ are assigned to epoxy (brown). The epoxy peak shows a broader width, which can be explained by the structural variations that epoxy develops when cured in the presence of other components, creating long-range interphases as shown in the second sample. A larger portion of the measuring points with values in the range of 13 nN < *F*_attr_ < 23 nN show a mixed behavior and cannot be assigned with certainty to either epoxy or PC. In this interphase, we distinguish between an epoxy-dominated interphase (yellow markers in the *k*_r_/*F*_attr_ diagram) and a PC-dominated interphase (light blue markers in *k*_r_/*F*_attr_ diagram). In the range of 6 nN < *F*_attr_ < 10 nN, the measuring points cannot be assigned to either boehmite or to PC (turquoise markers in *k*_r_/*F*_attr_ diagram). This seems to be an interphase/mixed behavior of boehmite NPs and PC. This is confirmed by the *k*_r_ values fanned out across the diagram. In the epoxy-dominated interphase (yellow markers) *k*_r_ appears to be constant, which is a behavior that we have already seen in the second model system for epoxy. Conversely, in the PC-dominated interphase (light blue markers) *k*_r_ shows a transitioning linear behavior from PC-typical stiffness to an epoxy-typical stiffness. We therefore conclude that the correlation between *k*_r_ and *F*_attr_ is not a simple linear correlation, in fact, it depends on the chemical structure.

In order to confirm this assessment, the structural assignments need to be transferred back to the spatial domain. By categorizing all the measuring points in the property domain as material phases or interphases, we achieve a manual principal component analysis. By transferring the mPCA to the spatial domain, the distribution of the components in the ternary composite in Figure 8b can be finally visualized. From this representation, it is clearly seen that the smoother part in the center consists of PC (blue) dissolved in the epoxy matrix (brown). In addition, boehmite NPs (green) form rougher structures; however, they are not released into the epoxy matrix since they are enclosed in every case by PC (blue). The points which were assigned to be interphases in Figure 7d can be found at the borders of the main components, representing the interphases of the nanocomposites.

## Conclusion

In this study we introduced and used two analytical tools which can significantly improve force spectroscopy measurements. First, we demonstrated the merit of a statistical approach of spatially resolved force spectroscopy data. Second, we demonstrated that by giving up the spatial information of a force map (spatial domain) we gain information about the property distribution (property domain). By taking advantage of this statistical approach, we showed that the maximum attractive force *F*_attr_ of any given force curve strongly depends on the chemical species present in the measured volume. The high specificity of this parameter was demonstrated with a high reproducibility for four different materials: two organic compounds, PC as a thermoplastic and epoxy as a thermoset, and two inorganic materials, glass and boehmite (γ-AlOOH), with up to four (epoxy) and three (boehmite) different sample–tip pairings. We also have a very strong indication that this parameter has a certain sensitivity for subsurface structures (model sample epoxy/PC). Consequently, by sensing into some depth of the sample, possible impurities on the sample surface or on the tip have less influence on *F*_attr_. This is unexpected since similar approaches, such as ncAFM and cAFM, rely heavily on clean surfaces. It is not clear yet if this difference is due to either investigating soft organic materials (with ncAFM smooth inorganic crystalline samples are preferred) or using *F*_attr_ instead of *F*_adh_ as in the cAFM case. *F*_adh_ takes into account the separation between the tip and the sample upon contact, which might be drastically changed due to impurities. We found that surface cleaning, which is common for mechanical measurements (microtome cut without further cleaning steps) is sufficient in order to get meaningful results. Also, a water layer from ambient conditions does not seem to pose a problem. In fact, *F*_attr_ is specific enough to assign a sufficient number of measurement points to one component, allowing for a manual principle component analysis. Retransferring the results of the mPCA into the spatial domain allows for a chemical mapping of the sample that is independent from the parameters describing the mechanical behavior. This was shown for two different force spectroscopy methods: force–distance curves (static) and intermodulation AFM (high-resolution, dynamic method) and validated by means of AFM-IR. By correlating different parameters obtained from single force curves in the property domain (e.g., *k*_r_/*F*_attr_ diagrams) a complete structure–property correlation can be achieved. In future studies, by correlating more than two parameters, a fully automated PCA of AFM force spectroscopy data can be pursued.

## Experimental

All force spectroscopy measurements were performed with a MFP-3D Asylum instrument (Oxford Instruments Asylum Research Inc., Santa Barbara, CA, USA). AFM FDCs were recorded with a frequency of 1 Hz. The spring constant of the cantilever was determined by a noninvasive thermal noise method. In the case of FDC experiments, the tip radius was estimated by fitting the reference measurements of glass and by applying the Hertz theory, as described in Supporting Information File 1. The AFM probes used were: Tip A (sQube, CP-NCH-SiO_A Nanoandmore, Wetzlar, Germany) with *k*_c_ = 38 N/m and *R* = 2 µm; tip B (HQ: NSC35, Mikromasch, Wetzlar, Germany) with *k*_c_ = 14.2 N/m and *R* = 23 nm; tip C (Pointprobe plus, PPP-NCHR, Nanosnesors Neuchâtel, Switzerland) with *k*_c_ = 51 N/m and *R* = 230 nm.

For high-resolution force spectroscopy an additional device was used in the AFM setup, an ImAFM (Intermodulation Products AB, Segersta, Sweden). The AFM probe used in this case was tip E (HQ: NSC35, Mikromasch, Wetzlar, Germany) with a resonance frequency *f*_0_ = 190 kHz, a spring constant *k*_c_ = 12 N/m, and a quality factor *Q* = 421.

The AFM-IR data were obtained using a NanoIR2s (Bruker/Anasys Instruments) coupled with a multichip quantum cascade laser (QCL) source (MIRcat, Daylight Solutions; with a tunable repetition rate in the range of 0–500 kHz and a spectral resolution of 0.1 cm^−1^) covering the spectral range from 900 cm^−1^ to 1900 cm^−1^. An Au-coated silicon probe (tip D) was employed.

The force curves were analyzed by SOFA [45] and the deconvolution of histograms was done by using Omnic (ThermoFisher Scientific) and fityk software [46].

The following epoxy system was used as the organic matrix material: bisphenol A diglycidyl ether (DGEBA, Araldite^®^ LY 556, Huntsman) cured with an anhydride curing agent, methyl tetrahydrophtalic acid anhydride (MTHPA, Aradur^®^ HY 917, Huntsman) and accelerated by an amine, 1-methyl-imidazole (DY070, Huntsman). The mixture of epoxy, hardener, and accelerator used was 100:90:1 w/w/w, respectively. As an additional organic component, bisphenol A polycarbonate from Makrolon 3108 (Goodfellow, UK) with *M*_w_ ≈ 49,550 g/mol and *M*_n_ ≈ 21,400, as measured by gel permeation chromatography (GPC), was used. As an inorganic component, boehmite (γ-AlOOH) from two different sources was used. For the second sample, boehmite was hydrothermally synthesized from elementary Al. The hydrothermal route is described elsewhere [16,37]. For the third sample, commercially available boehmite NP (HP14, Sasol) with an average primary size of 20 nm was acquired. The boehmite NP surface was modified with taurine (coverage of ≈16%) which was found to enable the subsequent electrospinning process. The nanoparticles were mixed in a solution of methylene chloride (CH_2_Cl_2_) and PC. The solution was electrospun to form fibers at 30 kV and at distance of 10 cm from the collector. The obtained nanocomposite fiber mat contained 20 wt % of taurine-modified boehmite NPs (Supporting Information File 1, Figure S2). The mat was embedded in the epoxy system and cured at 80 °C for 4 h, followed by a post-curing process at 120 °C for another 4 h. The sample was cut with a microtome for subsequent AFM measurements.

## Supporting Information

Additional information regarding the attractive regime and *A*_Ham_ under ambient conditions, the derivation of *k*_ref_ and *k*_r_, and the procedure for establishing the tip radius, *R*. Additional data treatment for the model sample, epoxy/PC, and an SEM micrograph of PC/BNP fibers (used in the epoxy/PC/BNP composite) is given.

File 1 Bulk chemical composition contrast.

## References

[1] Butt H-J, Cappella B, Kappl M (2005). Surf Sci Rep.

[2] Silbernagl D, Cappella B (2009). Surf Sci.

[3] Cappella B, Silbernagl D (2008). Thin Solid Films.

[4] Munz M, Sturm H, Schulz E, Hinrichsen G (1998). Composites, Part A.

[5] Ebeling D, Solares S D (2013). Beilstein J Nanotechnol.

[6] RosaZeiser A, Weilandt E, Hild S, Marti O (1997). Meas Sci Technol.

[7] Pakzad A, Simonsen J, Yassar R S (2012). Compos Sci Technol.

[8] Huang H, Dobryden I, Thorén P-A, Ejenstam L, Pan J, Fielden M L, Haviland D B, Claesson P M (2017). Compos Sci Technol.

[9] Ghasem Zadeh Khorasani M, Silbernagl D, Platz D, Sturm H (2019). Polymers (Basel, Switz).

[10] Platz D, Forchheimer D, Tholén E A, Haviland D B (2013). Beilstein J Nanotechnol.

[11] Chang J M, Guan X Y, Chen Y, Fan H J (2016). Polym Chem.

[12] Sharma S K, Sudarshan K, Pujari P K (2016). Phys Chem Chem Phys.

[13] Zare Y (2016). Composites, Part A.

[14] Park Y T, Qian Y Q, Chan C, Suh T, Nejhad M G, Macosko C W, Stein A (2015). Adv Funct Mater.

[15] Ebeling D, Eslami B, Solares S D J (2013). ACS Nano.

[16] Ghasem Zadeh Khorasani M, Elert A-M, Hodoroaba V-D, Agudo Jácome L, Altmann K, Silbernagl D, Sturm H (2019). Nanomaterials.

[17] Topolniak I, Hodoroaba V-D, Pfeifer D, Braun U, Sturm H (2019). Materials.

[18] Chung J, Munz M, Sturm H (2007). Surf Interface Anal.

[19] Cano Murillo N, Ghasem Zadeh Khorasani M, Silbernagl D, Hahn M B, Hodoroaba V-D, Sturm H (2020). J Appl Polym Sci.

[20] Krotil H-U, Stifter T, Waschipky H, Weishaupt K, Hild S, Marti O (1999). Surf Interface Anal.

[21] Höppener C, Schacher F H, Deckert V (2020). Small.

[22] Fu W Y, Zhang W (2017). Small.

[23] Guggisberg M, Bammerlin M, Loppacher C, Pfeiffer O, Abdurixit A, Barwich V, Bennewitz R, Baratoff A, Meyer E, Guntherodt H-J (2000). Phys Rev B.

[24] Sugimoto Y, Pou P, Abe M, Jelinek P, Pérez R, Morita S, Custance Ó (2007). Nature.

[25] Gross L, Mohn F, Moll N, Liljeroth P, Meyer G (2009). Science.

[26] Frisbie C D, Rozsnyai L F, Noy A, Wrighton M S, Lieber C M (1994). Science.

[27] Grundmeier G, Stratmann M (2005). Annu Rev Mater Res.

[28] Salerno M, Dante S (2018). Materials.

[29] Damircheli M, Payam A F, Garcia R (2015). Beilstein J Nanotechnol.

[30] Almonte L, Colchero J (2017). Nanoscale.

[31] Schmutz J-E, Hölscher H, Ebeling D, Schäfer M M, Anczykowski B (2007). Ultramicroscopy.

[32] Gady B, Schleef D, Reifenberger R, Rimai D S (1998). J Adhes.

[33] Das S, Sreeram P A, Raychaudhuri A K (2007). Nanotechnology.

[34] Fronczak S G, Dong J N, Browne C A, Krenek E C, Franses E I, Beaudoin S P, Corti D S (2017). Langmuir.

[35] Fronczak S G, Browne C A, Krenek E C, Beaudoin S P, Corti D S (2018). J Colloid Interface Sci.

[36] Bhattacharya B, Michalchuk A A L, Silbernagl D, Rautenberg M, Schmid T, Feiler T, Reimann K, Ghalgaoui A, Sturm H, Paulus B (2020). Angew Chem, Int Ed.

[37] Fankhänel J, Silbernagl D, Ghasem Zadeh Khorasani M, Daum B, Kempe A, Sturm H, Rolfes R (2016). J Nanomater.

[38] Butt H-J, Graf K, Kappl M (2003). Physics and Chemistry of Interfaces.

[39] Israelachvili J N (2011). Intermolecular and Surface Forces.

[40] Parsegian V A (2005). Van der Waals Forces : A Handbook for Biologists, Chemists, Engineers, and Physicists.

[41] Hamaker H C (1937). Physica (Amsterdam).

[42] Ghasem Zadeh Khorasani M, Silbernagl D, Szymoniak P, Hodoroaba V-D, Sturm H (2019). Polymer.

[43] Szymoniak P, Pauw B R, Qu X T, Schonhals A (2020). Soft Matter.

[44] Lam D C C, Chong A C M (2000). Mater Sci Eng, A.

[45] Silbernagl D, Schlesier T, Fritsche S, Sturm H J Open Res Software.

[46] Wojdyr M (2010). J Appl Crystallogr.

