# Supplementary material for: Bulk chemical composition contrast from attractive forces in AFM force spectroscopy
Source: Beilstein J Nanotechnol. 2021 Jan 18;12:58–71. doi: 10.3762/bjnano.12.5 (PMC7849247; doi:10.3762/bjnano.12.5)
Supplement: File 1 — Bulk chemical composition contrast. [file Beilstein_J_Nanotechnol-12-58-s001.pdf]

BEILSTEIN JOURNAL OF NANOTECHNOLOGY

Supporting Information

for

Bulk chemical composition contrast from attractive forces in AFM force spectroscopy

Dorothee Silbernagl, Media Ghasem Zadeh Khorasani, Natalia Cano Murillo, Anna Maria Elert and Heinz Sturm

Beilstein J. Nanotechnol. **2021,** *12,* 58–71. doi:10.3762/bjnano.12.5

Bulk chemical composition contrast

License and Terms: This is a supporting information file under the terms of the Creative Commons Attribution License (https://creativecommons.org/licenses/by/4.0). Please note that the reuse, redistribution and reproduction in particular requires that the author(s) and source are credited and that individual graphics may be subject to special legal provisions.
The license is subject to the *Beilstein Journal of Nanotechnology* terms and conditions: (https://www.beilstein-journals.org/bjnano/terms)

Attractive regime and A_{Ham} in ambient conditions

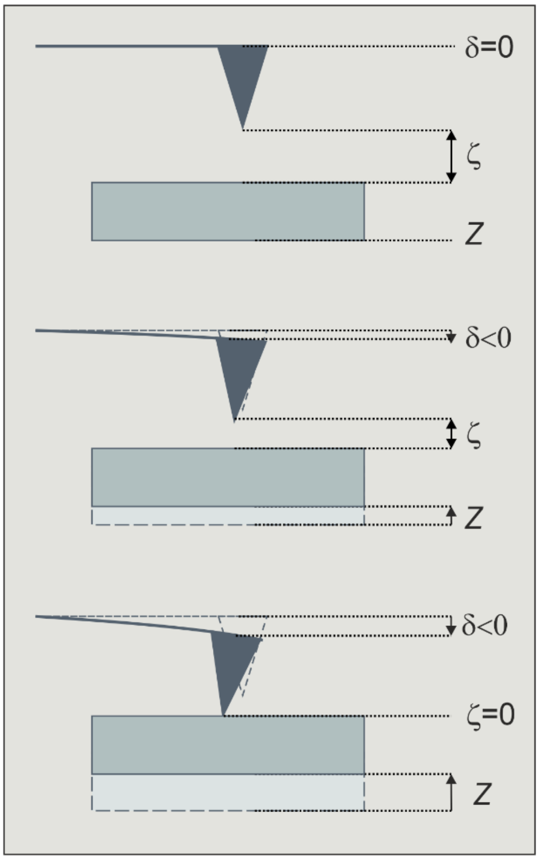

Figure S1 During approach of tip and sample two opposite movements have to be considered: The Z-piezo displacement *Z* and the cantilever deflection δ. This results in the net distance between the tip and the sample ζ, with δ and *Z* <0

$$\zeta = \delta - Z$$

The jump to contact is complete when ζ=0.

In order to estimate the Hamaker constant A_{Ham} of a given system, the force $F(\zeta)$ between the tip and the sample is given by:

$$F = k_c \delta = -\frac{A_{Ham}}{6}\frac{R}{\zeta^2} \quad \text{(S1)}$$

with spring constant k_c and tip radius *R*.

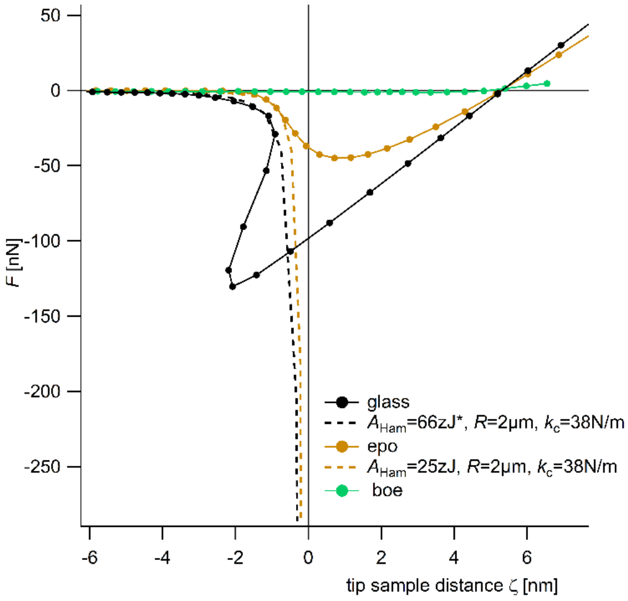

Figure S2 Since the experimental setup is defined for FDCs, shown in Figure 1 and Figure 2 (tip A, k_c=38 N/m, *R*=2µm and tip material SiO_2), the force *F* for glass, epoxy, and boehmite samples can be plotted as a function of the distance ζ between the tip and the sample and fitted with Equation S1.

Two problems become apparent: Especially for the measurement of glass, the tip–sample distance does not monotonically decrease. During the approach, the deflection

δ towards the sample is bigger than the gap between the sample surface and the equilibrium position of the cantilever. This is most probably due to the ambient water film which first contributes to the sample height, but shows no resistance when a force is applied by the tip. The second problem is to correctly find the point ζ = 0. In this case, A_{Ham} for SiO_2 as a function of SiO_2 in ambient condition is known from the literature [1] and the experimental curve was shifted along the *x*-axis to accommodate the theoretical curve. By applying the same shift to the experimental curves of epoxy and boehmite, we were able to fit A_{Ham} for epoxy as a function of SiO_2 in ambient condition. Although this fit yielded a reasonable value, this needs to be confirmed in a future work. Attractive forces between boehmite and SiO_2 are so small that fitting was not possible.

Repulsive regime and derivation of k_r and k_{eff}

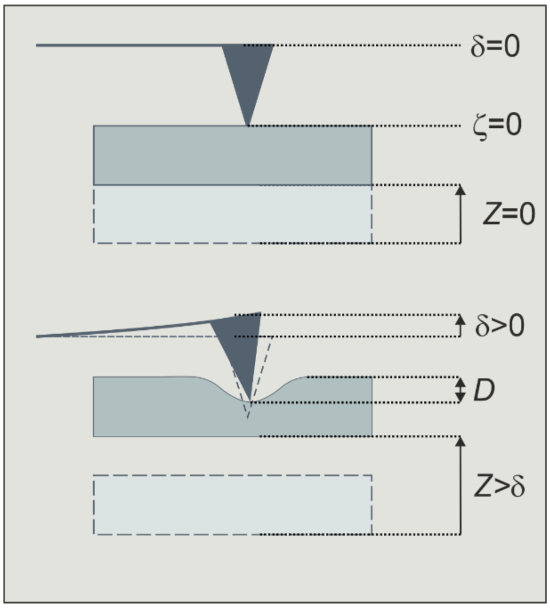

Figure S3 The AFM cantilever is assumed to behave like an elastic spring:

$$F = k_C\delta \qquad \text{(S2)}$$

with force *F*, spring constant k_c and deflection δ.

When the tip and the sample are in equilibrium during contact, the deformation *D* can be calculated by the difference between the piezo displacement *Z* and deflection δ:

$$D = Z - \delta \qquad \text{(S3)}$$

Additionally, the sample is assumed to have an elastic behavior with a spring constant *k*. When the tip and the sample are in contact, the forces causing a deflection δ and a deformation *D* are in balance.

$$k_c|\delta| = k|D| \quad \text{(S4)}$$

From Equation S3 and Equation S4, the following relationship between the piezo displacement Z and the deflection δ can be shown as:

$$kZ = kD + k\delta$$

with S4 $kZ = k_c\delta + k\delta$

$$\delta(k_c + k) = kZ$$

$$\delta = \frac{k}{k_c+k} Z = k_r Z \quad \text{(S5)}$$

This is not to be confused with the effective spring constant k_{eff} [2], which is the actual spring constant of the probed material by using Equation S2:

$$F = k_c\delta = \frac{k_c k}{k_c+k} Z = k_{eff} Z \quad \text{(S6)}$$

We prefer to work with k_r instead of k_{eff} since making use of the known spring constant k_c ($k_r \cdot k_c = k_{eff}$) suggests a comparability between measurements done with different cantilevers/tips. This is not the case. For comparison, the geometry of the tip has to be additionally taken into account. This is usually done by using the proper theoretical model describing the contact between elastic bodies (Hertz, DMT or JKR) [1].

Establishing the tip radius *R* for mechanical measurements

The Hertz's theory [3] takes the tip radius R into account. Hence, the geometry of the contact between the probe and the sample is defined and the reduced modulus E^* can be deduced from the deformation D (S3) in dependence on the applied force F (S2):

$$D = \left(\frac{F}{\sqrt{R}E^*}\right)^{2/3} \quad \text{(S7)}$$

Knowing the mechanical properties of the AFM tip (E_{tip} and ν_{tip}), the Young's modulus of the sample can be calculated as:

$$\frac{1}{E^*} = \frac{3}{4}\left(\frac{1-\nu_{tip}^2}{E_{tip}} + \frac{1-\nu^2}{E}\right) \quad \text{(S8)}$$

The reference measurements on a glass substrate can be used to establish the tip radius R. Since the mechanical properties of glass are well known (E_{glass} = 72 GPa, ν_{glass} = 0.3) the tip radius can be deduced by Equation S7 as the fitting equation.

Model sample I: data treatment

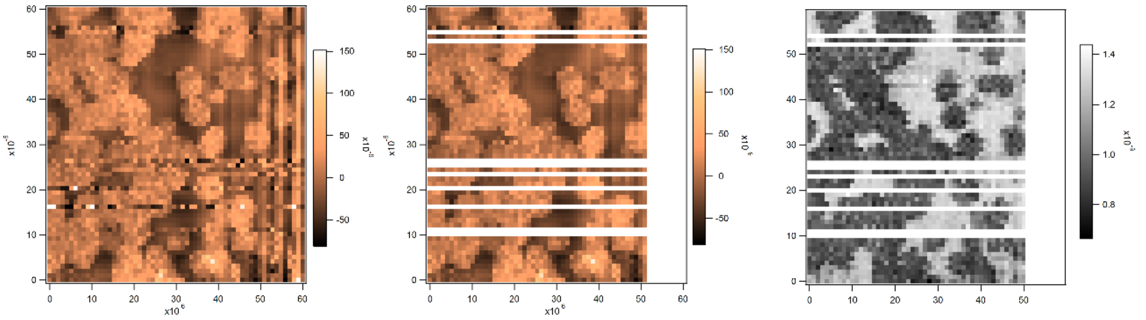

Figure S4: AFM FDC topography (left), topography (masked, middle), and F_{attr} (masked, right).The sample surface showed horizontal artefacts from the microtome cut. The right portion of the sample showed a distorted topography, also due to sample preparation. For further analysis, lines and columns were neglected as shown.

Epoxy/PC/boehmite composite

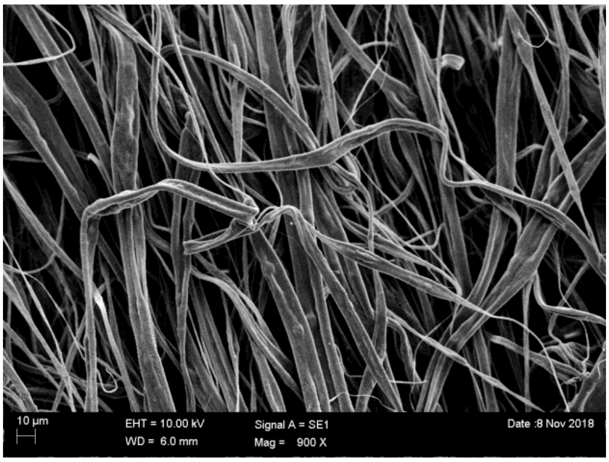

Figure S5: SEM image of electrospun PC fibers containing 20 wt % of boehmite NP (taurine modified HP14).

References

1. Das, S.; Sreeram, P. A.; Raychaudhuri, A. K. *Nanotechnology* **2007,** *18,* 035501. doi:10.1088/0957-4484/18/3/035501
2. Butt, H. J.; Cappella, B.; Kappl, M. *Surf. Sci. Rep.* **2005,** *59,* 1–152. doi:10.1016/j.surfrep.2005.08.003
3. Hertz, H. *J. Reine Angew. Math.* **1882,** *92,* 156–171. doi:10.1515/crll.1882.92.156
